# Axial N Ligand‐Modulated Ultrahigh Activity and Selectivity Hyperoxide Activation over Single‐Atoms Nanozymes

**DOI:** 10.1002/advs.202205681

**Published:** 2022-11-29

**Authors:** Han‐Chao Zhang, Pei‐Xin Cui, Dong‐Hua Xie, Yu‐Jun Wang, Peng Wang, Guo‐Ping Sheng

**Affiliations:** ^1^ CAS Key Laboratory of Urban Pollutant Conversion Department of Environmental Science and Engineering University of Science and Technology of China Hefei 230026 China; ^2^ Department of Civil & Environmental Engineering The Hong Kong Polytechnic University Kowloon Hong Kong 999077 China; ^3^ Key Laboratory of Soil Environment and Pollution Remediation Institute of Soil Science Chinese Academy of Sciences Nanjing 210008 China

**Keywords:** axial nitrogen, Fe(IV)=O, hyperoxide, micropollutant, single‐atoms nanozymes

## Abstract

Learning and studying the structure–activity relationship in the bio‐enzymes is conducive to the design of nanozymes for energy and environmental application. Herein, Fe single‐atom nanozymes (Fe‐SANs) with Fe–N_5_ site, inspired by the structure of cytochromes P450 (CYPs), are developed and characterized. Similar to the CYPs, the hyperoxide can activate the Fe(III) center of Fe‐SANs to generate Fe(IV)=O intermediately, which can transfer oxygen to the substrate with ultrafast speed. Particularly, using the peroxymonosulfate (PMS)‐activated Fe‐SANs to oxidize sulfamethoxazole, a typical antibiotic contaminant, as the model hyperoxides activation reaction, the excellent activity within 284 min^−1^ g^−1^_(catalyst)_ mmol^−1^_(PMS)_ oxidation rate and 91.6% selectivity to the Fe(IV)=O intermediate oxidation are demonstrated. More importantly, instead of promoting PMS adsorption, the axial N ligand modulates the electron structure of FeN_5_ SANs for the lower reaction energy barrier and promotes electron transfer to PMS to produce Fe(IV)=O intermediate with high selectivity. The highlight of the axial N coordination in the nanozymes in this work provides deep insight to guide the design and development of nanozymes nearly to the bio‐enzyme with excellent activity and selectivity.

## Graphical abstract

Learning the structure–activity relationship of bio‐enzymes is conducive to the design of nanozymes. Herein, Fe single‐atom nanozymes (Fe‐SANs) with Fe–N_5_ site, inspired by the structure of cytochromes P450 (CYPs), are developed for the hyperoxide activation to degrade micropollutants, and the crucial role of axial N for the activity and selectivity of Fe‐SANs is demonstrated.

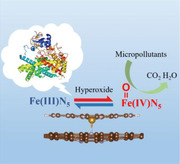

## 1 Introduction

Bio‐enzyme with optimized structure and ultrahigh efficiency is one of the presents to humans from nature, while the high cost and unstable structure in the complex environment are the main bottlenecks to seriously restrict its application. To solve this problem, the nanozymes, both have bio‐enzyme characteristics and high stability, loose operation conditions, and low cost,^[^ ^1^, ^2^, ^3^ ^]^ are developed to promote their practical application.^[^ ^4^ ^]^ Since iron oxide nanoparticles first showed enzyme‐like activity for the peroxidase decomposition,^[^ ^5^ ^]^ various nanozymes containing carbon materials,^[^ ^6^ ^]^ noble metals,^[^ ^7^ ^]^ and metal‐organic frameworks^[^ ^8^ ^]^ have been developed for biosensing,^[^ ^9^ ^]^ disease diagnosis,^[^ ^10^ ^]^ therapy,^[^ ^11^, ^12^ ^]^ and environmental remediation.^[^ ^13^ ^]^ However, the activity of nanozymes is still far below that of bio‐enzymes because of the low atom utilization efficiency,^[^ ^14^, ^15^ ^]^ and the inhomogenous composition of nanozymes results in its poor selectivity during the catalysis process.^[^ ^16^ ^]^ Up to now, there is still a long way to learn from enzyme structure for the rational design of nanozymes to gain higher activity and selectivity.

Cytochromes P450 (CYPs) are a significant superfamily of enzymes and wildly existed in the organisms of nature. The active site of CYPs contains a heme–iron center (Figure S1, Supporting Information), and the iron is tethered with five heteroatoms (N or S) in the horizontal plane and axial direction.^[^ ^17^ ^]^ The heme–iron center of CYPs can be activated by hyperoxide from Fe(III) to Fe(IV)=O intermediate for the oxidation of steroids, fatty acids, xenobiotics, and clearance of various compounds,^[^ ^18^, ^19^, ^20^ ^]^ which is crucial in the metabolism process. Furthermore, other bio‐enzymes like horseradish peroxidase and catalase with high activity for the hyperoxide decomposition also have a similar structure that iron tethered with four N in the horizontal plane and one N or S ligand in the axial direction.^[^ ^21^, ^22^ ^]^ These bio‐enzymes in microorganisms play a crucial role in the geochemical cycle and pollutant transformation. Thus, it is reasonable to hypothesize that this coordination structure is beneficial for the catalysis of hyperoxide decomposition for environmental application. However, the previously reported nanozymes still have great differences from CYPs in the structure and mechanism, thereby hindering the breakthrough in cognitive for the CYPs nanozymes.

Recently, some research revealed that single‐atoms catalysts (SACs)^[^ ^23^, ^24^, ^25^, ^26^, ^27^, ^28^ ^]^ are the ideal candidate for the nanozymes because the atomically dispersed metal centers in SACs exhibit a high catalytic activity via maximized atomic utilization efficiency and active sites density.^[^ ^29^, ^30^ ^]^ Furthermore, the homogenous structure of metal atoms in SACs is an excellent platform to deeply understand the structure–activity relationships at the atomic level. The previous study^[^ ^31^ ^]^ developed single iron atoms nanozymes (SANs) and proved their effectiveness for the O_2_ activation in biosensing and biomedical. Meanwhile, the CYPs could also be activated by the hyperoxide for the Fe(IV)=O intermediate generation, which has ultrahigh reactivity to the contaminants. However, a few studies^[^ ^29^, ^31^ ^]^ about FeN_5_ SANs considered hydroxyl radicals rather than Fe(IV)=O as the main oxidation intermediate in the hyperoxide activation process, which is quite different from the CYPs. This greatly encourages us to develop SANs with similar coordination structures and oxidation mechanisms with CYPs at the atomic level to explore its property in hyperoxide activation. Meanwhile, most SACs for the hyperoxide decomposition in the previous study were M‐N_4_ (M = Fe, Co, et al.) structures,^[^ ^32^, ^33^, ^34^, ^35^, ^36^ ^]^ which were still facing low activity and confusing structure–mechanism relationships.^[^ ^37^ ^]^ The extra N coordination in the axial direction probably modulates the electronic density of single iron atoms, thereby enhancing the catalytic efficiency of Fe‐SANs close to the bio‐enzymes. Thus, developing Fe‐SANs with FeN_5_ sites inspired by CYPs’ structure would scale new heights for the hyperoxide decomposition activity and selectivity in the environmental field.

In this work, we prepared Fe–N_5_ SANs with similar structures and properties to the CYPs. The hyperoxide, especially peroxymonosulfate (PMS), can active Fe‐SANs from Fe(III) to Fe(IV)=O intermediate, which have ultrahigh efficiency and selectivity to the oxidation of pollutants for water purification. Instead of radicals or singlet oxygen, the mediated electron transfer via the cycle of Fe(III) to Fe(IV)=O was the main mechanism in the PMS‐activated Fe‐SANs process, which is the same as CYPs. Intriguingly, instead of promoting of PMS adsorption, the crucial role of axial N coordination in the FeN_5_ site for the lower reaction energy barrier and promoted electron transfer to hyperoxide was demonstrated by the theoretical calculation at the atomic level. The highlight of the axial N coordination of Fe‐SANs in this work greatly helps us to understand the structure–activity relationship of Fe–N_5_ sites in the CYPs and Fe‐SANs, which is instructive for the further development of SANs according to this rational design in the energy and environmental application.

## 2 Results and Discussion

### 2.1 Preparation and Characterization of SANs

**Figure 1:**
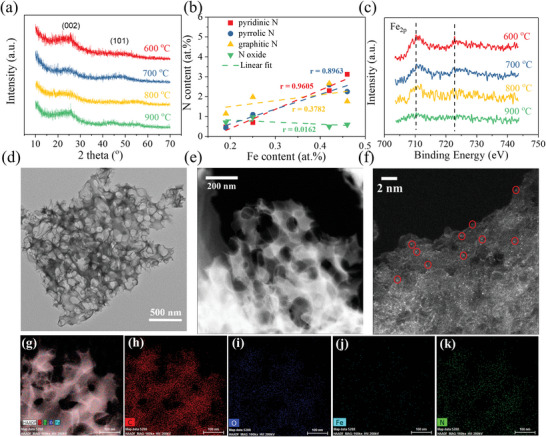
Characterization of Fe‐SANs. a) The XRD patterns; b) correlation of Fe and N contents in Fe‐SANs prepared at various pyrolysis temperatures. c) Fe 2p high‐resolution XPS spectra; d) TEM image and e,f) HAADF‐STEM images of Fe‐SANs‐800 °C. The red cycles in (f) were the single Fe atoms on the carbon substrate; g,h) element mappings of Fe‐SANs‐800 °C.

Using the phenanthroline‐Fe and nano‐MgO as the precursor and template, respectively, the Fe‐SANs under different pyrolysis temperatures (600, 700, 800, 900 °C) were synthesized and characterized. **Figure** **1**a shows no obvious peaks of Fe species in the X‐ray diffraction (XRD) patterns of Fe‐SANs, indicating that there are no Fe particles in the Fe‐SANs.^[^ ^38^ ^]^ In the Raman spectrum (Figure S2, Supporting Information), the ratio of the D band (1350 cm^−1^) to the G band (1575 cm^−1^) of Fe‐SANs increased with the rise of pyrolysis temperature, implying more defects of the Fe‐SANs under higher temperature. Meanwhile, the specific surface area of Fe‐SANs increased at the initial and then decreased with the increase of pyrolysis temperature (Figure S3, Supporting Information), and Fe‐SANs‐800 °C had the highest specific surface area (1325.5 m^2^ g^−1^). The X‐ray photoelectron spectroscopy (XPS) survey (Figure S4, Supporting Information) of Fe‐SANs shows the content of C increased while those of N, O, and Fe gradually decreased with the rise of pyrolysis temperature. Furthermore, the N 1s spectra of Fe‐SANs (Figure S4, Supporting Information) could be deconvoluted into pyridinic N (398.2 eV), pyrrolic N (399.6 eV), graphitic N (400.9 eV), and nitric oxide (NO*_x_*, 402.0 eV)^[^ ^39^ ^]^ (Figure S5, Supporting Information). It was noted that only the content of pyridinic N was strongly correlated with the content of Fe under different pyrolysis temperatures (Figure 1b), probably due to the Fe atoms being coordinated with the pyridinic N in the Fe‐SANs. Meanwhile, two peaks located at 710.2 (Fe 2p_3/2_) and 723.5 eV (Fe 2p_1/2_) in the high‐resolution Fe 2p XPS spectrum indicate the Fe(III) species in the Fe‐SANs^[^ ^40^ ^]^ (Figure 1c). It is worth noting that only the surface Fe (<10 nm) on the SANs could be detected by the XPS,^[^ ^39^ ^]^ while all the Fe in the SANs could be measured by the inductively coupled plasma‐atomic emission spectrometry (ICP‐AES). When the pyrolysis temperature was higher than 800 °C, the measurement results of Fe content by the ICP‐AES were nearly equal to that by the XPS (Figure S6, Supporting Information), which means most of the Fe atoms were loading on the surface of Fe‐SAN at high pyrolysis temperatures.

Transmission electron microscopy (TEM) images show the porous carbon structure of Fe‐SANs, and the pore size ranged from 50 to 200 nm (Figure 1d and Figure S7a–c, Supporting Information). More importantly, a high density of Fe single atoms on the carbon substrate was directly observed (Figure 1e,f) by the aberration‐corrected high‐angle annular dark‐field scanning transmission electron microscopy (HAADF‐STEM), which convincingly evidenced the abundant single Fe atoms in the Fe‐SANs‐800 °C. Moreover, the elemental mapping of Fe‐SANs‐800 °C shows a homogenous distribution of Fe and N elements on the carbon materials (Figure 1g–k). Nevertheless, both single atoms and clusters of Fe species were observed in the Fe‐SANs‐900 °C (Figure S8, Supporting Information), indicating that part of the Fe atoms were agglomerated under the highest pyrolysis temperature.

**Figure 2:**
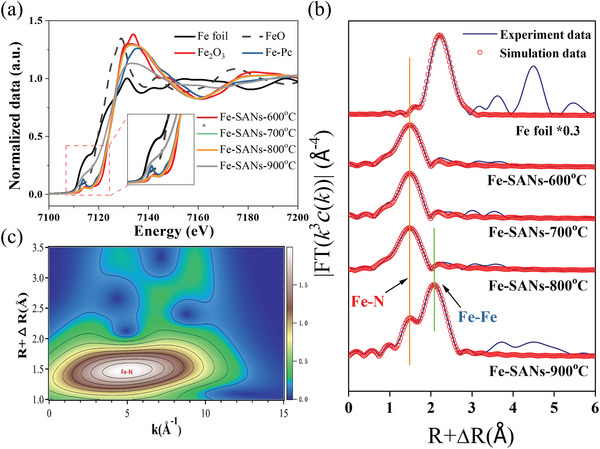
Atomic structure and chemical state characterization of Fe–N_5_. a) Normalized Fe K‐edge XANES; b) Fourier transform extended X‐ray absorption fine structure (FT‐EXAFS) of Fe‐SANs under different pyrolysis temperatures; c) the wavelet transforms of EXAFS for the Fe‐SANs‐800 °C.

The state and fine coordination structure of Fe atoms in the Fe‐SANs were further characterized by the extended X‐ray absorption fine structure (EXAFS). In the Fe K‐edge X‐ray absorption near edge structure (XANES) spectra (**Figure** **2**a), the energy absorption threshold of the Fe‐SANs (600, 700, 800 °C) was the same as Fe_2_O_3_, indicating the valence state of iron was Fe(III). Furthermore, the consistent K‐edge XANES spectrum with phthalocyanine iron (Fe‐Pc) implied that the square‐planar Fe–N_4_ moieties (D_4_h symmetry) existed in the Fe‐SANs.^[^ ^41^ ^]^ However, the pre‐edge peak of Fe‐SANs was much lower than that of Fe‐Pc (Figure 2a), implying the coordination structure of Fe‐SANs was not FeN_4_ (Fe‐Pc). Furthermore, the EXAFS fitting parameters revealed that the coordination numbers of Fe in the SANs (600, 700, 800 °C) were ranged from 5.4 to 5.6 (>4) (Table S3, Supporting Information). The extra coordination numbers than 5 were attributed to the ‐OH on the Fe–N_5_ site,^[^ ^42^ ^]^ and other O species were mainly located on the Fe‐SANs substrate as the hydroxyl (‐OH) and carbonyl (C=O) functional groups. Thus, the FeN_5_ including four N coordination in the horizontal plane and one N coordination in the axial direction was the main coordination unit in the Fe‐SANs.^[^ ^43^ ^]^ In the Fourier transform extended X‐ray absorption fine structure (FT‐EXAFS, Figure 2b), a strong peak of Fe—N located at 1.50 Å was observed in the spectrum of Fe‐SANs. Meanwhile, the Fe—Fe scattering path located at 2.1 Å was only emerged in the Fe‐SANs‐900 °C spectrum (Figure 2b). That meant the Fe atoms were agglomerated to generate some clusters at a pyrolysis temperature of 900 °C, which was consistent with the HAADF‐STEM images. The wavelet transformed (WT) extended EXAFS spectra of Fe‐SANs were obtained with a high resolution in the *R* spaces (Figure 2c and Figure S9, Supporting Information). Only a single WT peak at ≈5.0 Å^−1^ attributed to the Fe−N coordination could be observed in the Fe‐SANs‐800 °C (Figure 2c), which further demonstrated the five N coordination of Fe atoms in the horizontal and axial directions.

### 2.2 Hyperoxide‐Activated Fe‐SANs for Micropollutant Removal

**Figure 3:**
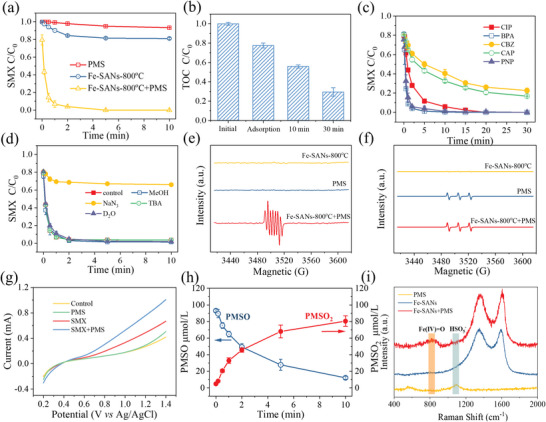
Performance and mechanism for the PMS‐activated Fe‐SANs to degrade micropollutants. a) SMX degradation kinetics by PMS‐activated Fe‐SANs system; b) total organic carbon (TOC) removal after adsorption by the Fe‐SANs‐800 °C and oxidation of PMS‐activated Fe‐SANs‐800 °C for 10 and 30 min. c) The ciprofloxacin (CIP), bisphenol A (BPA), carbamazepine (CBZ), chloramphenicol (CAP), and p‐nitrophenol (PNP) degradation kinetics in the PMS‐activated Fe‐SANs‐800 °C system; d) effect of different radicals scavengers and D_2_O on the SMX degradation in the PMS‐activated Fe‐SANs system; e,f) electron paramagnetic resonance spectra of PMS‐activated Fe‐SANs system by using the e) DMPO and f) TEMP as the trapping agent; g) linear sweep voltammetry obtained by the Fe‐SANs‐800 °C electrode in the presence of PMS and SMX; h) the kinetics of PMSO degradation and PMSO2 production in the PMS‐activated Fe‐SANs system. i) Raman spectra of PMS, Fe‐SANs‐800 °C, and its reaction systems. Reaction conditions: [Fe‐SANs‐800 °C] = 100 mg L^−1^, [PMS] = 1 mmol L^−1^, [SMX] = 10 mg L^−1^, [MeOH] = [TBA] = 100 mmol L^−1^, [NaN_3_] = 10 mmol L^−1^, [DMPO] = 5.0 mmol L^−1^, [TEMP] = 15 mmol L^−1^, [PMSO] = 100 µmol L^−1^, pH 7.0.

The CYPs can catalyze hyperoxide decomposition with the cycle of Fe(III) to Fe(IV)=O intermediate.^[^ ^17^, ^43^ ^]^ In this work, Fe‐SANs also had a high catalysis activity for the hyperoxide decomposition such as hydrogen peroxide, peroxydisulfate, and PMS (Figure S10, Supporting Information). Among these hyperoxides, the PMS‐activated Fe‐SANs showed the highest efficiency for the degradation of sulfamethoxazole (SMX), a widely detected antibiotic in the aquatic environment. Only 9% of SMX was directly oxidized by the PMS alone within 10 min under the same condition (**Figure** **3**a). Moreover, without adding PMS, only 20% SMX was adsorbed by the Fe‐SANs‐800 °C. However, after adding PMS to the Fe‐SANs‐800 °C solution, all the SMX was degraded completely in less than 5 min (Figure 3a), which was 254 times faster than that of PMS direct oxidation, and this performance was also quite better than those for the Fe^2+^ ions, *α*‐Fe_2_O_3_, and phthalocyanine iron (Fe‐Pc) under the same conditions (Figure S11, Supporting Information). That meant the PMS could activate Fe‐SANs to generate reactive oxidation species for SMX removal with ultrahigh efficiency. It is also found that the SANs prepared at a higher pyrolysis temperature would result in a higher SMX degradation rate (Figure S12a, Supporting Information). With the rising of pyrolysis temperature, the graphitization degree of Fe‐SANs increased, resulting in the stronger hydrophobic and *π*–*π* interaction with micropollutants, which might be helpful for micropollutant adsorption and electron transfer with Fe‐SANs for its degradation. Based on the characterization, both the Fe single atoms and clusters existed in the Fe‐SANs‐900 °C. To avoid the potential impact of Fe clusters, the Fe‐SANs‐800 °C was used in the following experiment unless specified. The Fe‐SANs‐800 °C maintained a stable performance for the SMX degradation in the acid and neutral pH, but gradually dropped at the alkaline solution (Figure S12b, Supporting Information). With the PMS dosage increased from 0.1 to 2.0 mmol L^−1^, the pseudo‐first‐order kinetic constant of SMX degradation rate was increased from 0.27 to 2.53 min^−1^ (Figure S13a, Supporting Information). Meanwhile, the SMX degradation rate increased from 0.05 to 3.69 min^−1^ with the Fe‐SANs‐800 °C dosage increased from 10 to 200 mg L^−1^ (Figure S13b, Supporting Information). More importantly, 47% of SMX was mineralized after 10 min reaction in this Fe‐SANs system (Figure 3b), and this value further increased to 76% after 30 min reaction. Besides SMX, the Fe‐SANs also gave good performances for other pollutants degradation (Figure 3c). The degradation rates of the phenolics, like bisphenol A (BPA), p‐nitrophenol (PNP), and ciprofloxacin (CIP) were higher than those of carbamazepine (CBZ) and chloramphenicol (CAP) (Figure 3c), indicating this Fe‐SANs system had a high activity for the removal of pollutants with electron‐rich groups. Compared to the previous M‐N_4_ (M = Fe, Co, et al.) SACs in the literature, the PMS‐activated FeN_5_ SANs in this work exhibit a higher efficiency (284 min^−1^ g^−1^_(catalyst)_ mmol^−1^_(PMS)_) for micropollutant degradation (Table S4, Supporting Information), supported the unique structure of Fe–N_5_ with axial N coordination in Fe‐SANs is beneficial for hyperoxide activation to degrade micropollutants.

### 2.3 Mechanism of PMS‐Activated Fe‐SANs

In the conventional PMS activation process, the radicals (SO_4_^•−^/•OH) or singlet oxygen (^1^O_2_) is the main oxidation species.^[^ ^44^ ^]^ However, in this work, adding 100 mmol L^−1^ of methyl alcohol (MeOH) or tertiary butyl alcohol (TBA) to quench the SO_4_^•−^/•OH and •OH^[^ ^40^ ^]^ had no influence on the SMX degradation by the PMS‐activated Fe‐SANs‐800 °C (Figure 3d), indicating that the •OH or SO_4_^•−^ did not exist in this system. Although the addition of NaN_3_, as a scavenger of ^1^O_2_,^[^ ^45^ ^]^ greatly suppressed the degradation of SMX in this Fe‐SANs system (Figure 3d), it was mainly due to the PMS being directly reduced by the NaN_3_.^[^ ^46^ ^]^ Meanwhile, using the D_2_O as a solvent to extend the lifetime of ^1^O_2_ has no promotion to the SMX degradation (Figure 3d), which meant ^1^O_2_ had less role in this Fe‐SANs catalysis process. Moreover, the electron paramagnetic resonance experiment was conducted to directly determine the main oxidation species in this process. Using the 5,5‐dimethyl‐1‐pyrroline‐N‐oxide (DMPO) as the trapping agent of •OH and SO_4_^•−^, no peaks were identified in the PMS or carbon solution (Figure 3e). However, in the PMS‐activated Fe‐SANs solution, a series of strong peaks denoted as the DMPOX were observed (Figure 3e), which meant other oxidation species existed in this system but were not due to the production of •OH or SO_4_^•−^.^[^ ^47^ ^]^ For the 2,2,6,6‐tetramethyl‐4‐piperidinol (TEMP) to trap ^1^O_2_ (Figure 3f), the signal of TEMP‐^1^O_2_ adduct, 2,2,6,6‐tetramethylpiperidine‐N‐oxyl (TEMPO), was emerged in the sole PMS solution. Meanwhile, the signal of TEMPO was also observed in the PMS‐activated Fe‐SANs solution (Figure 3f), but it had no difference from the peak intensity with the PMS alone. That was due to the PMS could be directly decomposed to produce ^1^O_2_ slowly,^[^ ^48^, ^49^ ^]^ while the addition of Fe‐SANs to the PMS solution had no promotion for the ^1^O_2_ production. To further quantify the ^1^O_2_ production in these processes, the singlet oxygen sensor green (SOSG) was used as the specific probe to the ^1^O_2_ via generating green fluorescence (*E*_x_/*E*_m_: 504/525 nm).^[^ ^50^ ^]^ The fluorescence intensity in the sole PMS solution was gradually increased after adding the SOSG probe (Figure S14, Supporting Information), directly proving the continuous generation of ^1^O_2_ by the PMS decomposition. However, in the PMS‐activated Fe‐SANs system, a strong fluorescence signal was emerged but quenched quickly (Figure S14, Supporting Information). It was noted that the ^1^O_2_ was incapable to induce the quenching of SOSG fluorescence, thereby further verifying the ^1^O_2_ was not the reactive species in the PMS‐activated Fe‐SANs process.

### 2.4 Mediated Electron Transfer via the Cycle of Fe(III) and Fe(IV)=O Intermediate

The O_2_ or hyperoxide can activate the iron‐center of CYPs from Fe(III) to Fe(IV)=O, and the Fe(IV)=O intermediate pass its oxygen along to the substrate with remarkable speed.^[^ ^51^ ^]^ That is one of the main functions of CYPs, as the monooxygenases, for the oxidation of the substrate.^[^ ^52^ ^]^ In this work, the above results demonstrated the radicals or ^1^O_2_ were not the oxidation species in the PMS‐activated Fe‐SANs process. Thus, it is reasonable to hypothesize the mediated electron transfer via the cycle of Fe(III) and Fe(IV)=O intermediate was the main mechanism for the PMS‐activated Fe‐SANs system, and the Fe(IV)=O intermediate was the critical reactive oxidation species for the micropollutant removal. To measure the electron transfer between Fe‐SANs and micropollutant in this process, the Fe‐SANs‐800 °C loading on the fluorine‐doped tin oxide electrode was prepared for the linear sweep voltammetry (LSV) and *i–t* test. In the LSV curves, the current was a little increased after adding SMX or PMS to the electrolyte individually (Figure 3g), but this current on the Fe‐SAN‐800 °C loading electrode was greatly increased after adding SMX and PMS to the electrolyte simultaneously. Meanwhile, the *i–t* curve also revealed that the PMS greatly enhanced the electron transfer of SMX to the Fe‐SANs (Figure S15, Supporting Information). These results directly supported the PMS greatly promoting the electron transfer between Fe‐SANs with micropollutants, and the electron transfer was the main nonradicals pathway for micropollutant degradation in this system. Furthermore, to study the main electron transfer site of Fe‐SANs, the KSCN was used to cover the iron atoms on the SANs because of its strong binding capacity to the iron atoms.^[^ ^53^ ^]^ With the dosage of KSCN increased from 0.1 to 1.0 mmol L^−1^, the SMX degradation rate gradually decreased from 0.675 to 0.039 min^−1^ (Figure S16, Supporting Information). These results revealed that the single iron atoms on the Fe‐SANs were the main electron shuttle for the redox of PMS and micropollutant, and thus the cycle of Fe(III) to the Fe(IV)=O intermediate in the Fe‐SANs was a possible mechanism. To further demonstrate the critical role of Fe(IV)=O intermediate in this Fe‐SANs system, the methyl phenyl sulfoxide (PMSO) chemical probe was conducted because Fe(IV)=O intermediate can selectively oxidize PMSO to produce methyl phenyl sulfone (PMSO_2_) via the oxygen atom transfer mechanism.^[^ ^54^ ^]^ The PMS and Fe‐SANs alone could not oxidize PMSO to generate PMSO_2_ (Figure S17, Supporting Information). In the PMS‐activated Fe‐SANs system, the PMSO_2_ product was detected by the ultra‐performance liquid chromatography‐tandem time of flight mass spectrometry (Figure S18, Supporting Information). Furthermore, the concentration of PMSO_2_ in the solution was gradually raised with the decrease of the PMSO concentration (Figure 3h), and the *η*(ΔPMSO_2_/ΔPMSO) achieved 91.6% after 10 min reaction. These results evidenced that the Fe(IV)=O intermediate was the main reactive oxidation species in this PMS‐activated Fe‐SANs system, and the selectivity of the oxygen transfer mechanism for the substrate oxidation in this system was achieved at 91.6%. However, the Fe(V)=O could also oxidize PMSO to the PMSO_2_ through oxygen transfer reaction, and the Fe(V)=O was widely regarded as the main reactive oxidation species in the Fe‐SACs‐activated PMS system.^[^ ^34^, ^36^, ^55^ ^]^ The Fe(IV)=O was produced by the one‐electron transfer reaction of oxygen to the Fe(III)—N_5_ while the Fe(V)=O was produced by the two‐electron transfer reaction. Furthermore, the heterolysis  of O—O bond would generate Fe(IV)=O (or denoted O=Fe(IV)‐Porp+•), but the homolysis of O—O (two electron transfer) would produce Fe(V)=O and hydroxyl radicals (Equations (1)–(3)).^[^ ^56^ ^]^ However, no radicals were detected in the Fe‐SANs‐activated PMS process (Figure 3d), which proved that Fe(IV)=O was the main reactive oxidation species. To directly measure the Fe(IV)=O in the reaction, the in situ Raman was conducted (Figure 3i). The peak located at 1060 cm^−1^ represents HSO_5_^−^ of PMS^[^ ^34^ ^]^ and the Fe‐SANs only show D (1350 cm^−1^) and G (1575 cm^−1^) bands in the Raman spectra. After adding PMS to the Fe‐SANs, a new peak at 815 cm^−1^ was observed due to the PMS‐activated Fe‐SANs. Furthermore, this peak was different from that of PMS adsorbed on the Fe center (Fe‐PMS*, at 843 cm^−1^).^[^ ^57^ ^]^ Thus, this new peak was probably attributed to the Fe (IV)=O, which was from the PMS‐activated Fe(III)—N_5_ center in the Fe‐SANs

(1)

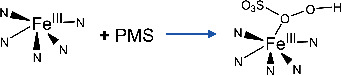



(2)

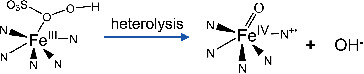



(3)

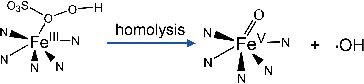




### 2.5 Density Functional Theory (DFT) Calculation

**Figure 4:**
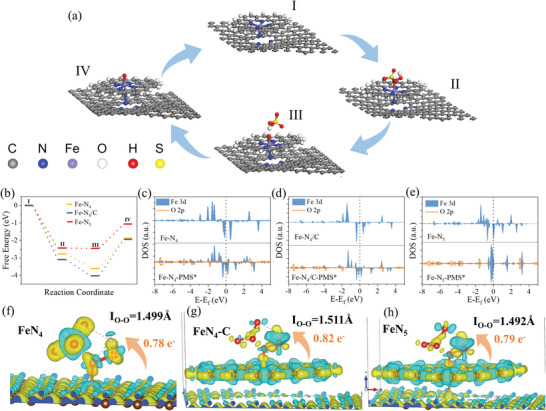
Theoretical investigation of PMS‐activated Fe‐SANs. a) Proposed reaction pathways for the PMS‐activated Fe‐SANs to generate Fe(IV) = O intermediate; b) free energy diagram for the PMS‐activated Fe–N_4_, Fe–N_4_/C, and Fe–N_5_ to generate Fe(IV) = O intermediate; c–e) PDOS of Fe, O atom of PMS adsorbed on the Fe center in the Fe–N_4_, Fe–N_4_/C, and Fe–N_5_ (*E*_f_ is marked in each graph with the black dashed line); charge density difference (*ρ*_total_ – *ρ*_substrate_ – *ρ*_PMS_) in optimized configurations of f) FeN_4_, g) Fe–N_4_/C, and h) FeN_5_. The isosurface contour is 0.015 e bohr^−1^.

Based on the above results, the reaction pathway of PMS‐activated Fe‐SANs was proposed (**Figure** **4**a). First, the PMS molecular was adsorbed on the Fe atoms site of Fe‐SANs to generate the complex of Fe‐SANs‐PMS*. Then the Fe(IV)=O intermediate was generated by the heterolytic cleavage of O—O bond in the PMS. Subsequently, the Fe(IV)=O intermediate transferred the oxygen atom to the micropollutant rapidly. After that, the Fe(IV)=O was reduced to Fe(III) for the further cycle of catalysis reaction, and this process is similar to the CYPs catalysis process. Recently, a few studies also considered the high valence iron‐oxo was the main oxidation species in the SACs‐activated PMS system.^[^ ^29^, ^51^, ^58^ ^]^ However, all of the iron coordination structures in these studies were Fe–N_4_, which lacked the axial coordination of N compared to the bio‐enzyme structure. Thus, the DFT calculation was used to demonstrate the role of axial N coordination in the FeN_5_ site for the Fe(IV)=O intermediate production. Three models, Fe–N_4_, FeN_4_ with carbon substrate (Fe–N_4_/C), and Fe–N_5_ SANs (Figure S19, Supporting Information) were developed for the DFT calculation, and the double layer model of Fe–N_4_ was conducted to avoid the potential impact of the extra carbon layer. First, the Bader charge analysis (Figure S20, Supporting Information) shows the charge of the Fe center was increased after it combined with carbon layer or extra N coordination, indicating the valence state of Fe was raised in the Fe–N_5_ SANs. Meanwhile, the higher spin state of Fe‐SANs was easier prone to high‐valent iron‐oxo species generation during the PMS activation.^[^ ^55^ ^]^ The adsorption energy calculation proved the FeN_5_ site has no superiority over the PMS adsorption, which is widely recognized as a key step in promoting PMS activation.^[^ ^34^, ^35^ ^]^ Furthermore, along the whole reaction pathway, the desorption of HSO_4_^−^ is the main reaction barrier for the Fe(IV)=O intermediate production (Figure 4b), and the FeN_5_ SANs shows a lower reaction barrier compared to the Fe–N_4_ and Fe–N_4_/C. That also revealed that the moderate adsorption energy for the PMS molecular adsorption is beneficial for HSO_4_^−^ desorption to generate Fe(IV)=O intermediate. Moreover, the density of states (DOS) of the Fe center and O atom of adsorbed PMS on the Fe sites were calculated. The d‐band center of Fe in the FeN_5_ site is farther to the Fermi‐level compared to the Fe–N_4_ and Fe–N_4_/C (Figure 4c–e), which is beneficial to the desorption of HSO_4_^−^ molecular.^[^ ^59^ ^]^ Furthermore, the d band of Fe–N_5_‐PMS* is narrow and close to the Fermi‐level (Figure 4e), and the projected density of states (PDOS) of Fe–N_5_ shows a higher overlap between the Fe 3d and O 2p orbitals than that of Fe–N_4_ and Fe–N_4_/C. That means the Fe center in the FeN_5_ SANs has higher interaction with O atom after PMS adsorption, thereby promoting the generation of Fe(IV)=O intermediate for the further oxidation of micropollutants. The charge density difference further shows the interfacial electron transfer number and structure of the surface‐bound PMS* (O—O bond length) for the FeN_5_ have little difference with FeN_4_, thus the axial N of FeN_5_ SANs did not promote the PMS adsorption (Figure 4f–h). However, a great difference in charge transfer density on the two sides of O—O bond emerged after PMS adsorbed on the Fe–N_5_, while it was homogenous after PMS adsorbed on Fe–N_4_ (Figure 4f,h). The difference in charge transfer density on the two sides of O—O bond is beneficial to its cleavage, and that further supported the axial N in the FeN_5_ SANs promoted the heterogenous cleavage of O—O bond for the Fe(IV)=O intermediate generation (Equation (2)). All of these calculations intrinsically demonstrated that the extra N coordination in the axial direction modulated the electronic density of single iron atoms, which greatly lowered the reaction barrier and promotes the electron transfer to the PMS for generating Fe(IV)=O intermediate. Therefore, compared to the previous M–N_4_ (M = Fe, Co, et al.) SACs, the PMS‐activated FeN_5_ SANs in this work exhibit a higher efficiency for micropollutant degradation (Table S4, Supporting Information).

### 2.6 Application of Fe‐SANs for Water Purification

The cycle experiment of Fe‐SANs showed the SMX degradation efficiency decreased from 100% to 83% at 10 min after the fifth run (Figure S21a, Supporting Information). Furthermore, the activity of Fe‐SANs was recovered by a thermal treatment (N_2_, 800 °C, 2 h) after the fifth run, and 100% SMX could be degraded within 5 min by using the regenerated Fe‐SANs (Figure S21a, Supporting Information). More importantly, less than 0.4% Fe ions of Fe‐SANs were leaching into the solution after reaction (lower than 15 µg L^−1^, Figure S21b, Supporting Information), which was much lower than the limitation of the standard of drinking water (300 µg L^−1^). Moreover, the unstable structure of conventional bio‐enzymes in the complex aquatic environment is one of the main bottlenecks to seriously restrict its application. However, our PMS‐activated Fe‐SANs still completely removed SMX at less than 15 min in the secondary effluent, Reservoir, and River water matrix (Figure S22, Supporting Information), implying the stable performance and great application potential of Fe‐SANs in the actual aquatic environment.

## 3 Conclusions

Herein, inspired by the CYPs structure, the Fe–N_5_ site SANs were developed and characterized in this work. Using the PMS‐activated Fe‐SANs to oxidize SMX as the model hyperoxides activation reaction, the excellent activity within 284 min^−1^ g^−1^_(catalyst)_ mmol^−1^_(PMS)_ oxidation rate and 91.6% selectivity to the Fe(IV)=O intermediate oxidation were demonstrated, which are similar to the CYPs. Particularly, the axial N ligand is beneficial to the lower barrier and promoted electron transfer to the PMS rather than promoting PMS adsorption, which is crucial for the Fe(IV)=O intermediate generation with high selectivity. In this work, the highlight of axial N in Fe–N_5_ SANs shines a light on the rational design of nanozymes, which is leaning from bio‐enzymes, for energy and environmental applications. Moreover, with a deeper understanding of the enzyme structure based on the rapid development of structural biology, it is reasonable to believe the SANs with higher activity and selectivity would be booming in further work.

## Conflict of Interest

The authors declare no conflict of interest.

## Supporting information

Supporting Information

## Data Availability

The data that support the findings of this study are available from the corresponding author upon reasonable request.
