# Supplementary material for: Axial N Ligand‐Modulated Ultrahigh Activity and Selectivity Hyperoxide Activation over Single‐Atoms Nanozymes
Source: Adv Sci (Weinh). 2022 Nov 29;10(3):2205681. doi: 10.1002/advs.202205681 (PMC9875630; doi:10.1002/advs.202205681)
Supplement: Supplementary file 1 — Supporting Information [file ADVS-10-2205681-s001.pdf]

Axial N Ligand modulated ultra-high activity and selectivity hyperoxide activation over Single-Atoms Nanozymes

Han-Chao Zhang[1,2], Pei-Xin Cui[3], Dong-Hua Xie[1], Yu-Jun Wang[3], Peng Wang[2], Guo-Ping Sheng[1]*

1. CAS Key Laboratory of Urban Pollutant Conversion, Department of Environmental Science and Engineering, University of Science and Technology of China, Hefei, 230026, China

2. Department of Civil & Environmental Engineering, The Hong Kong Polytechnic University, Kowloon, Hong Kong 999077, China

3. Key Laboratory of Soil Environment and Pollution Remediation, Institute of Soil Science, Chinese Academy of Sciences, Nanjing, 210008, China

***Correspondence author:** Guo-Ping Sheng, gpsheng@ustc.edu.cn

Materials and methods

Chemicals.

In this work, the $Fe(OAc)_2$, SMX, 5, 5-dimethyl-1-pyrroline-*N*-oxide (DMPO), and 2, 2, 6, 6-tetramethyl-4-piperidinol (TEMP) were purchased from J&K Scientific Ltd. SOSG was purchased from Meilun biotechnology Ltd., Dalian. The methyl phenyl sulfoxide (PMSO) and methyl phenyl sulfone ($PMSO_2$) were purchased from Aladdin Industrial Corporation. Singlet oxygen sensor green (SOSG) was purchased from Thermo Fisher Scientific. PMS, nano-MgO (100 nm), 1, 10-phenanthroline, and other chemicals were purchased from Shanghai Chemical Reagent Co., China.

Preparation of the Fe-SANs.

Briefly, iron (II) acetate (1 mmol) and 1, 10-phenanthroline (4 mmol) were added to the 100 mL ethanol and sonicated for 30 min. After that, 6 g nano MgO (100 nm) template were added to this mixture and then stirred (500 rpm) under reflux at 60oC for 12 h. Then, the ethanol was removed by the rotary evaporation and the remaining solid was dried by vacuum drying at 60°C. This solid was transferred to the tube furnace and heated to 600, 700, 800, and 900°C for 2 h under the nitrogen atmosphere with a ramp of 5 ^{o}C/min. The obtained carbon materials were washed with the 1 mol/L HNO_3 solution and deionized water 3 times to remove the nano MgO. At last, this carbon material was dried by vacuum drying at 60oC overnight to obtain the Fe-SANs and denoted as Fe-SAN-600°C, Fe-SAN-700°C, Fe-SAN-800°C, and Fe-SAN-900°C.

Catalysis experiment.

The batch experiment was conducted in a 100 mL beaker. Fe-SANs (10-200 mg/L) were added into a 25 mL SMX solution (10 mg/L) with stirring at 400 rpm. After the adsorption equilibrium (20 min), the PMS of various dosages (0.1-2.0 mmol/L) was added to the solution to start the reaction. At each time interval, 0.2 mL of the solution was taken out and filtered to remove Fe-SANs. The PMS in the filtrate was quenched with excess sodium thiosulfate before the SMX was quantified by high-performance liquid chromatography (HPLC). The pH of the reaction solution was controlled by the 200 mmol/L borate buffer. Besides SMX, the degradation of other micropollutants like ciprofloxacin (CIP), bisphenol A (BPA), chloramphenicol (CAP), p-nitrophenol (PNP), and carbamazepine (CBZ) were also conducted to prove the effectiveness of this Fe-SANs. In addition, MeOH (100 mmol/L) was dosed into the solution to scavenge $SO4^{\cdot -}$ and ·OH. To identify the role of $^1O_2$, NaN_3 (100 mmol/L) was dosed to scavenge the $^1O_2$, and D_2O was used as the solvent to extend the lifetime of $^1O_2$. The PMSO was also used to identify the Fe(IV)=O in this system.

DFT calculation.

The DFT calculation was performed by CASTEP code in Materials Studio. The GGA-PBE exchange-correlation potential and ultra-soft pseudopotentials were used during the calculation process. The energy cutoff was set to 400.0 eV and the 1 × 1 × 1 Monkhorst Pack mesh k-point was employed for surface calculations. The convergence tolerances were set to 2×10^{-5} eV per atom for energy, 2×10^{-3} Å for maximum displacement, and 0.05 eV $Å^{-1}$ for the maximum force during the geometry

optimization. The van der Walls interactions were considered by the DFT dispersion correction (DFT-D).

Characterization of Fe-SANs

The X-ray diffraction (XRD) patterns of the Fe-SANs were measured by a Philips X'Pert PRO SUPER diffractometer equipped with graphite monochromatized Cu Kα radiation (λ=1.541874 Å). The element content and valence states on the Fe-SANs surface were tested by X-ray photoelectron spectroscopy (XPS) using an ESCALAB250 (Thermo Fisher Inc., USA). The Fe content of SANs was measured by the Inductively Coupled Plasma-Atomic Emission Spectrometry (ICP-AES, Optima 7300 DV, PerkinElmer Inc. USA). The Raman spectrum of Fe-SANs was carried out using the LabRam HR Evolution instrument (HORIBA Jobin Tvon Co., France) with a 512 nm laser. The specific surface area of Fe-SANs was measured on a builder 4200 instrument (TriStar II 3020M, Micromeritics Instrument Co., USA). The sharp and structure of Fe-SANs were characterized with a transmission electron microscope (TEM) (H7650, Hitachi Co., Japan), and the loading state of Fe on the SANs was measured by the aberration-corrected high-angle annular dark-field scanning transmission electron microscopy (HAADF-STEM, JEM-ARM200F STEM, JEOL). Fe K-edge X-ray absorption fine structure (XAFS) spectra of the Fe-SANs were recorded at the BL14W1 beamlines of the Shanghai Synchrotron Radiation Facility (SSRF).

Linear sweep voltammetry (LSV) measurement.

For the measurement of electron transfer from Fe-SANs to the PMS and SMX, the LSV measurement was conducted at room temperature in a three-electrode configuration with an electrochemical work station (CHI 760E, Chenhua Instrument Co., China). The Fe-SANs-800°C were coated on the fluorinated tin oxide (FTO) glass as the working electrode. The Pt and the Ag/AgCl electrode were used as the counter and reference electrodes, respectively. A 200 mmol/L boric acid buffer was used as the electrolyte. The current at a working electrode was measured by increasing the potential from 0.2 to 1.4 V with a scan rate of 10 mV/s. Meanwhile, the *i*-*t* curves of Fe-SANs electrode were obtained at 0 V *vs*. Ag/AgCl.

Cycle experiment and actual surface water application.

To investigate the recyclability, Fe-SANs-800°C after the catalysis reaction was recovered by centrifugation, washed three times by the distilled water, and then used for further catalytic degradation of SMX repeatedly. After five cycles, a thermal treatment (N_2, 800°C, 2 h) was conducted for the regeneration of the Fe-SANs-800°C.

The SMX degradation experiment was also conducted using surface water like secondary effluent, reservoir water, and river water as the solution. The physical characteristics of surface water were provided in Table S1. All experiments were conducted in duplicates. The averaged data and standard deviation were presented.

Analytic methods

The concentration of H_2O_2 was determined using titanium oxysulfate solution via a spectrophotometry method at 410 nm. The concentrations of PDS and PMS during the experiments were measured by the KI spectrophotometry methods at 352 nm. The

concentrations of micropollutants and PMSO/$PMSO_2$ were analyzed using HPLC (Model 1260, Agilent Inc., US) equipped with a C_{18} column (4.6 mm ID × 250 mm, 5 μm particle size) and a UV detector. The details measurement condition were provided in Table S2. The PMSO/$PMSO_2$ in the PMS activated Fe-SANs solution was also detected by the Ultra Performance Liquid Chromatography-Tandem time of flight mass spectrometry (UPLC-TOF). The electron paramagnetic resonance (EPR) spectrum was conducted on a spectrometer (JES-FA200, JEOL Co., Japan) to detect the free radicals in this system. The total organic carbon (TOC) concentration was measured using a TOC analyzer (Muti N/C 2100, Analytik Jena, Germany).

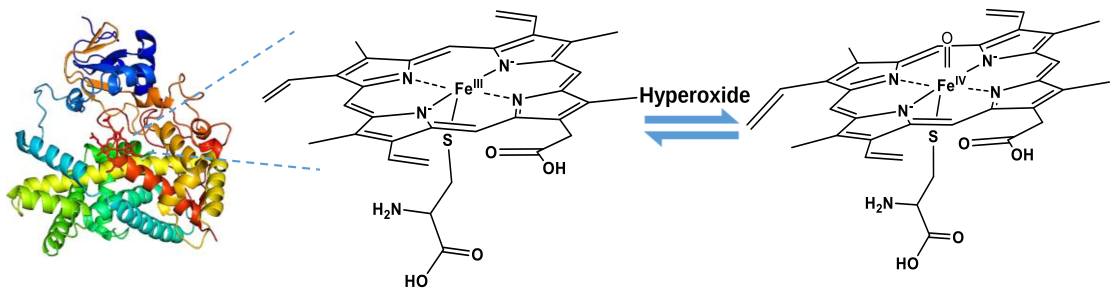

Figure S1. Structure of mammalian cytochrome P450 and its activation mechanism.

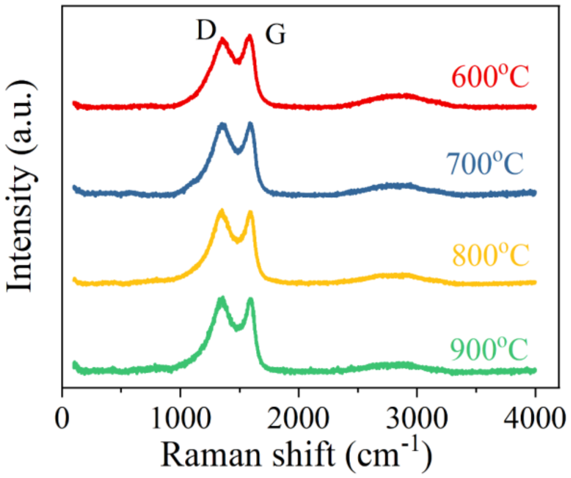

Figure S2. Raman spectra of Fe-SANs prepared at different pyrolysis temperatures.

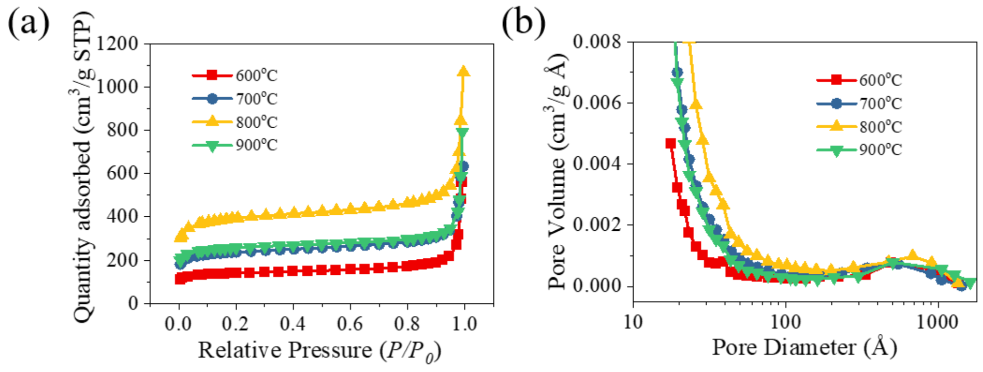

Figure S3. N_2 sorption isotherms (a); and pore size distributions (b) of Fe-SANs prepared at different pyrolysis temperatures.

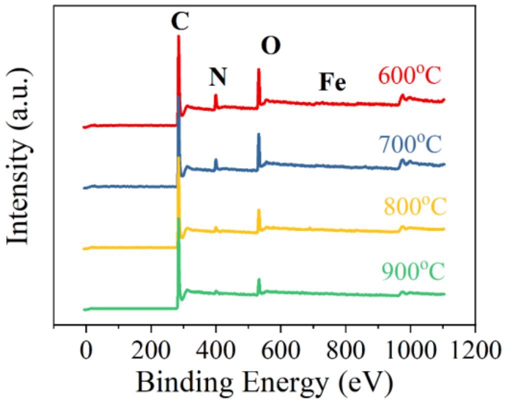

Figure S4. XPS survey of Fe-SANs prepared at different pyrolysis temperatures.

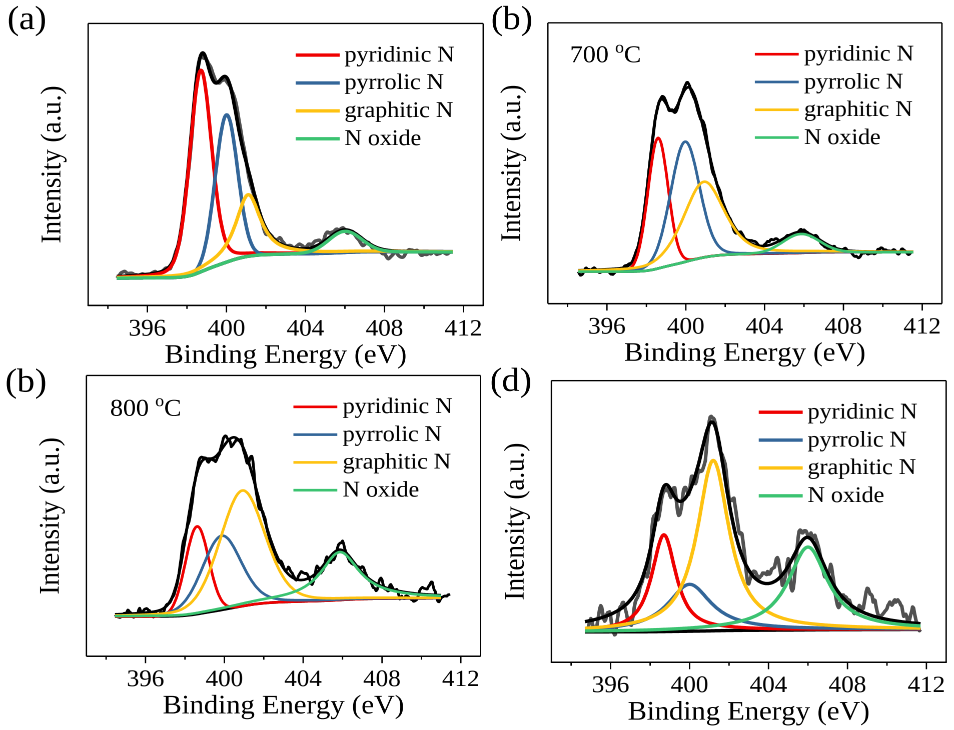

Figure S5. High resolution XPS N1s spectra of Fe-SANs prepared at different pyrolysis temperatures: 600°C (a); 700°C (b); 800°C (c); and 900°C (d).

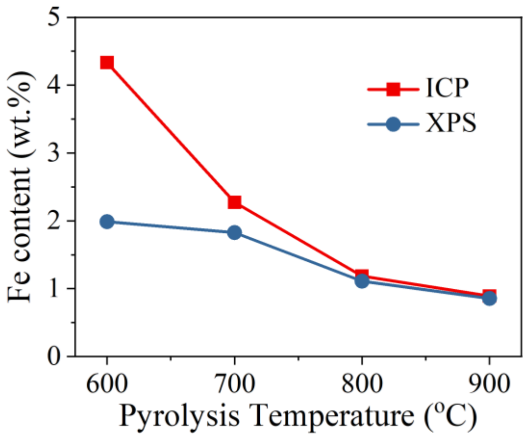

Figure S6. The measurement of Fe content in Fe-SANs prepared at different pyrolysis temperatures by XPS and ICP.

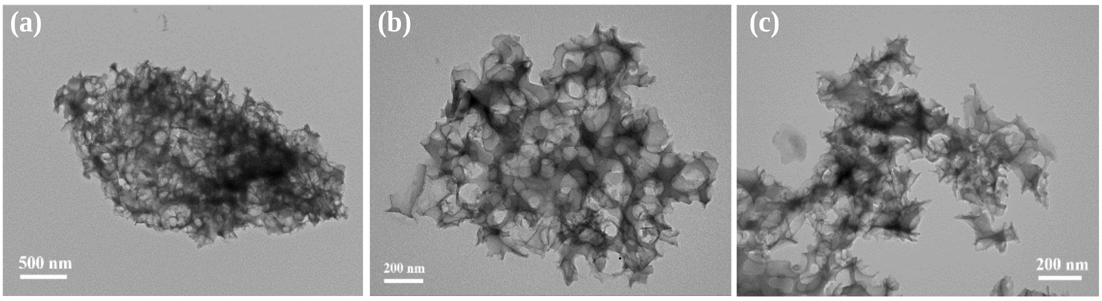

Figure S7. The TEM images of Fe-SANs-600°C (a); Fe-SANs-700°C; and Fe-SANs-900°C (c);

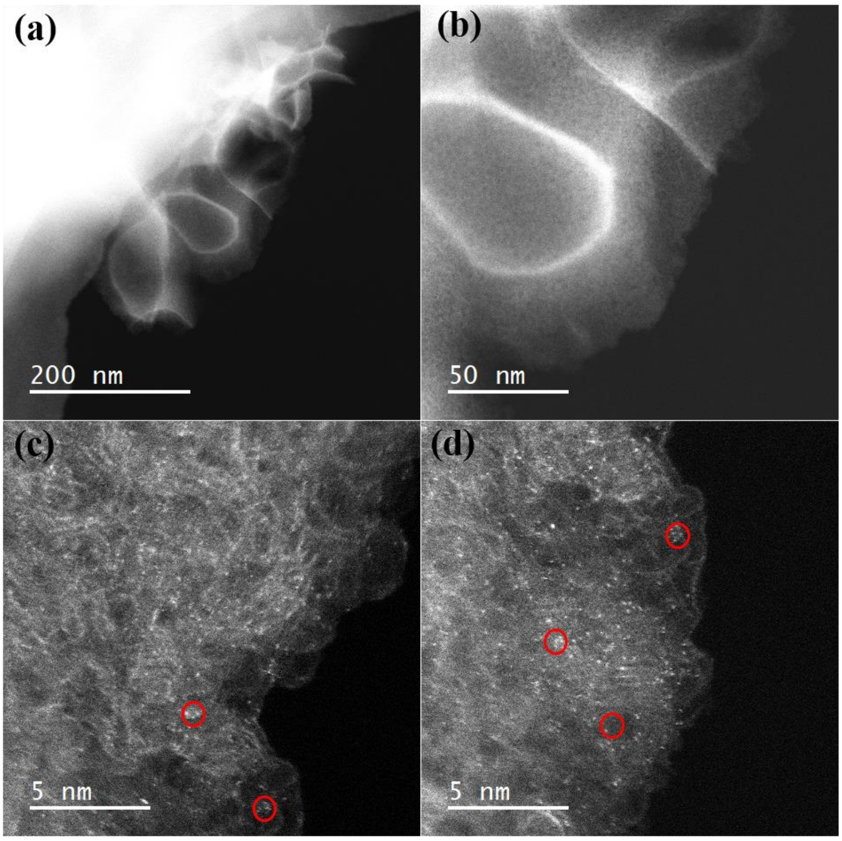

Figure S8. The HADDF-STEM images of Fe-SANs-900°C (a, b, c, d). The red cycles were the clusters on the Fe-SANs-900°C.

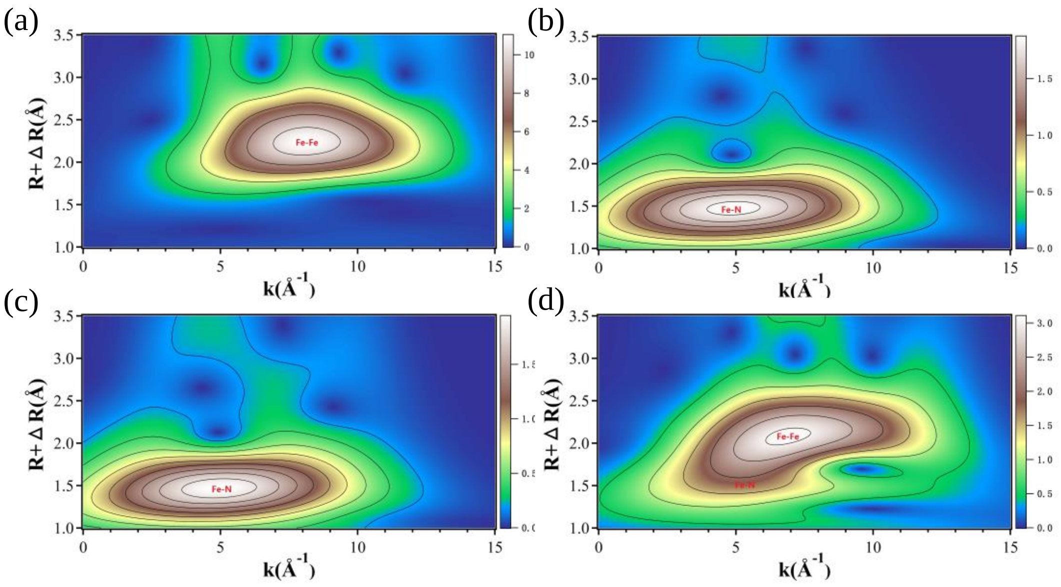

Figure S9. The wavelet transformed of EXAFS of Fe-foils (a); Fe-SANs-600°C (b); Fe-SANs-700°C (c); and Fe-SANs-900°C (d).

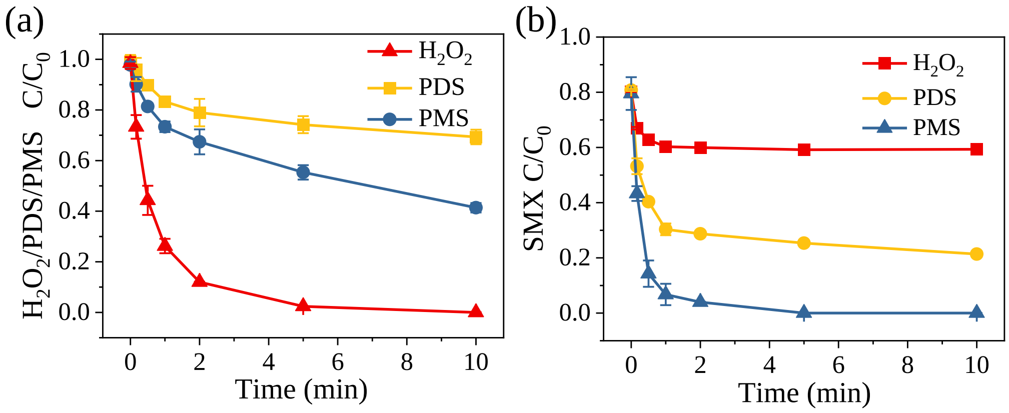

Figure S10. The H_2O_2/PDS/PMS decomposition kinetics (a) and SMX degradation kinetics in the H_2O_2/PDS/PMS activated Fe-SANs-800°C systems. Reaction condition: [SMX] = 10 mg/L, [H_2O_2/PDS/PMS] = 1 mmol/L, [Fe-SANs-800°C] = 100 mg/L, pH = 7.

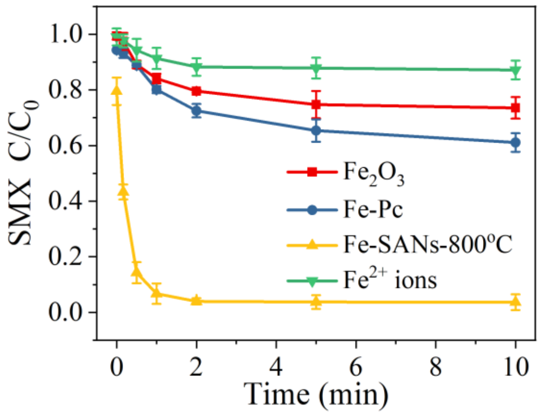

Figure S11. The SMX degradation kinetics in the Fe^{2+}, Fe_2O_3, phthalocyanine iron (Fe-Pc), and Fe-SANs-800°C activated PMS systems. Reaction condition: [SMX] = 10 mg/L, [PMS] = 1 mmol/L, [Fe^{2+}] = [Fe_2O_3] = [Fe(II)-Pc] = [Fe-SANs-800°C] = 100 mg/L, pH = 7.

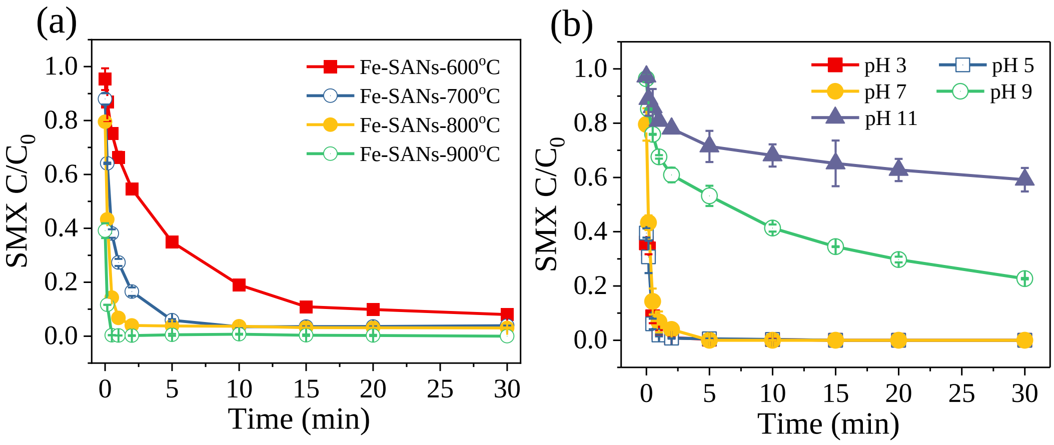

Figure S12. SMX degradation kinetics by the PMS activated Fe-SANs prepared at different pyrolysis temperatures (a); SMX degradation kinetics by the PMS activated Fe-SANs-800°C at different pHs. Reaction condition: [SMX] = 10 mg/L, [PMS] = 1 mmol/L, [Fe-SANs] = 100 mg/L, pH = 7 in (a).

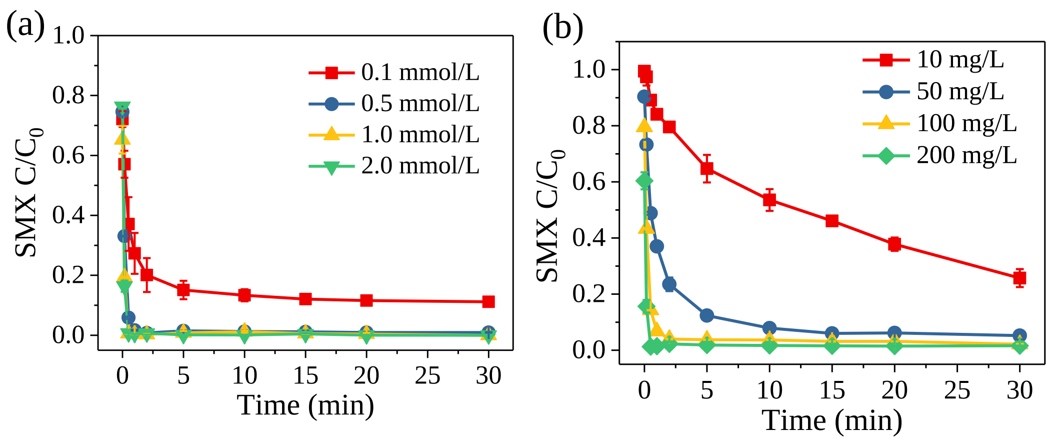

Figure S13. SMX degradation kinetics by the PMS activated Fe-SANs-800°C under different PMS dosages (a) and Fe-SANs-800°C dosages (b). Reaction condition: [SMX] = 10 mg/L, [PMS] = 1 mmol/L in (b), [Fe-SANs-800°C] = 100 mg/L in (a), pH = 7.

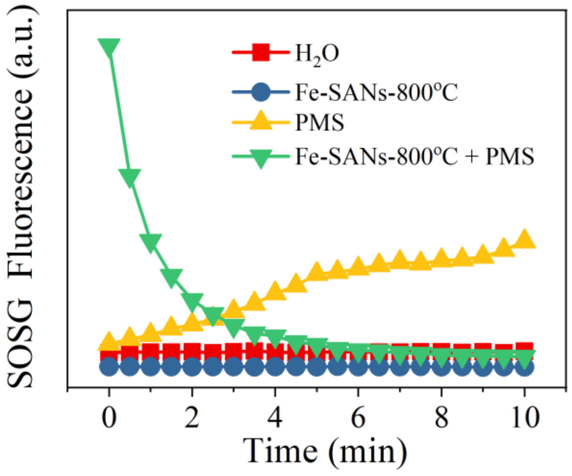

Figure S14. The fluorescence intensities of SOSG (E_x/E_m: 504/525 nm) in different PMS activated Fe-SANs-800°C systems. Reaction condition: [SMX] = 10 mg/L, [PMS] = 1 mmol/L, [Fe-SANs-800°C] = 100 mg/L, [SOSG] = 100 μmmol/L, pH = 7.

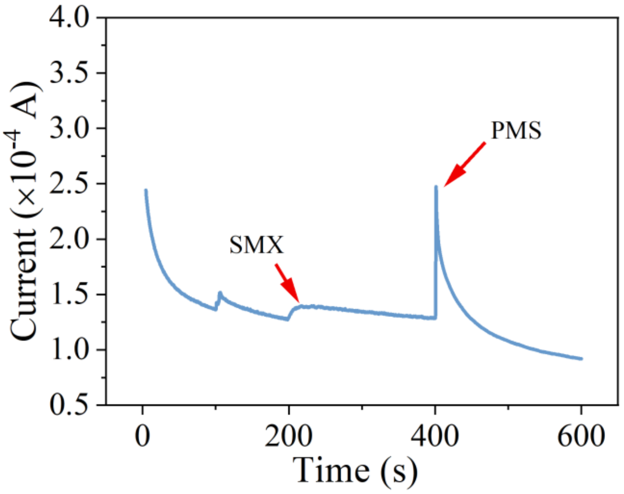

Figure S15. *I*-*t* curve obtained from Fe-SANs-800°C/FTO electrode at 0 V *vs* Ag/AgCl using 200 mmol/L borates as the electrolyte.

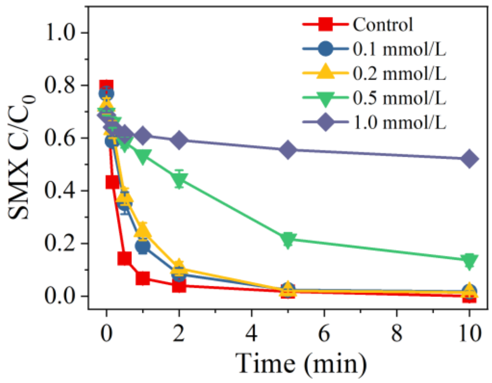

Figure S16. The inhibiting effect of gradient dosage of KSCN on the SMX degradation in the PMS activated Fe-SANs-800°C system.

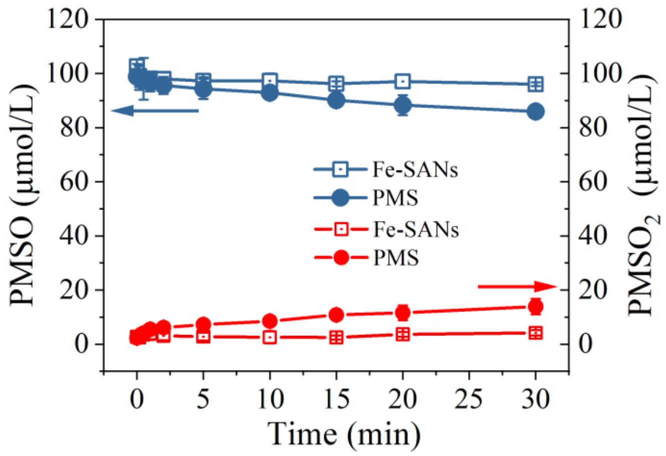

Figure S17. The degradation of PMSO and formation of $PMSO_2$ by the PMS and Fe-SANs-800°C alone. Reaction condition: [Fe-SANs-800°C] = 100 mg/L, [PMS] = 1 mmol/L, [PMSO] = 100 μmol/L, pH 7.0.

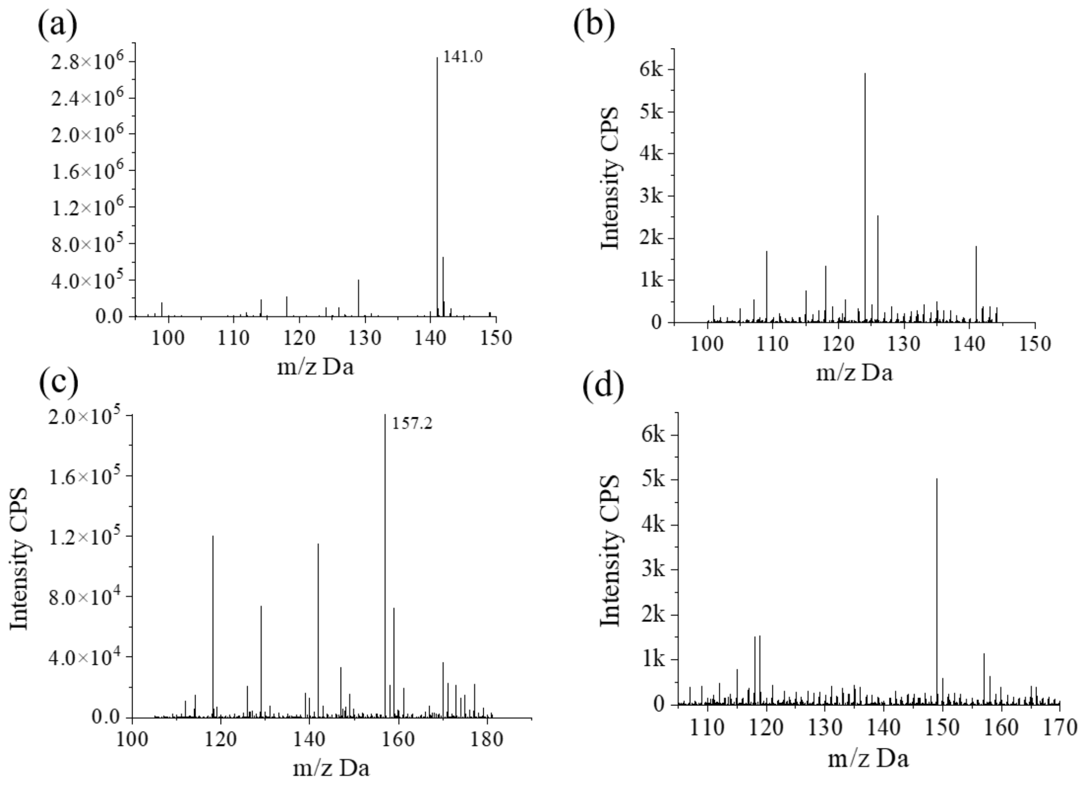

Figure S18. The MS (a) and MS-MS (b) spectra of PMSO; MS (c) and MS-MS (d) spectra of $PMSO_2$ in the PMS activated Fe-SANs system.

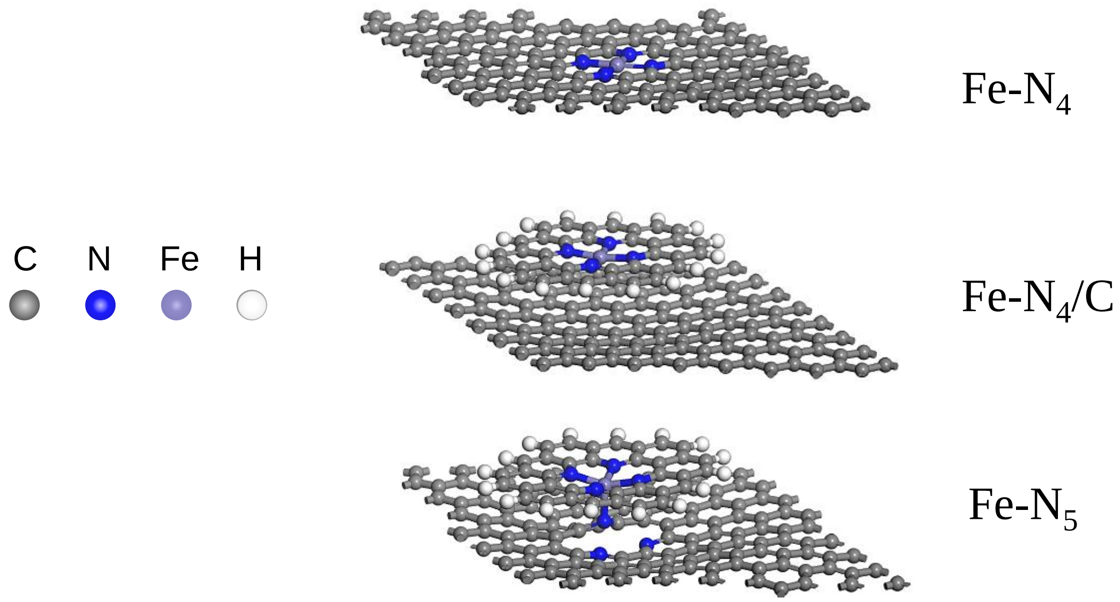

Figure S19. Theoretical models of Fe-N_4, Fe-N_4/C, and Fe-N_5 SANs.

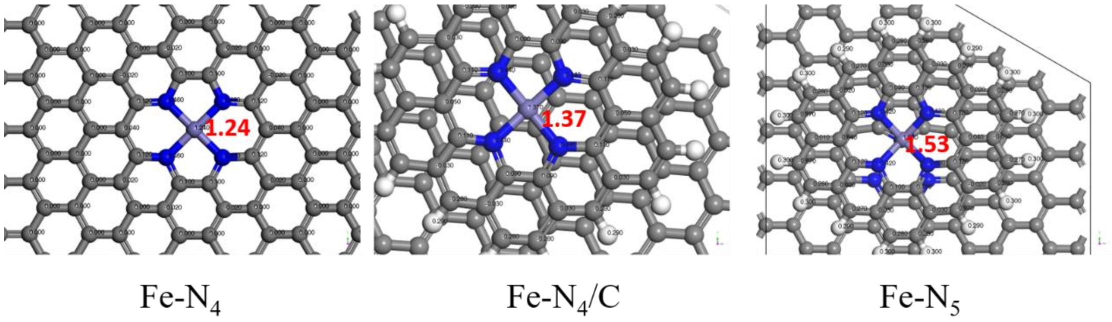

Figure S20. Bader charges of Fe-N_4, Fe-N_4/C, Fe-N_5 SANs for describing the distribution of electrons in chemical bonds.

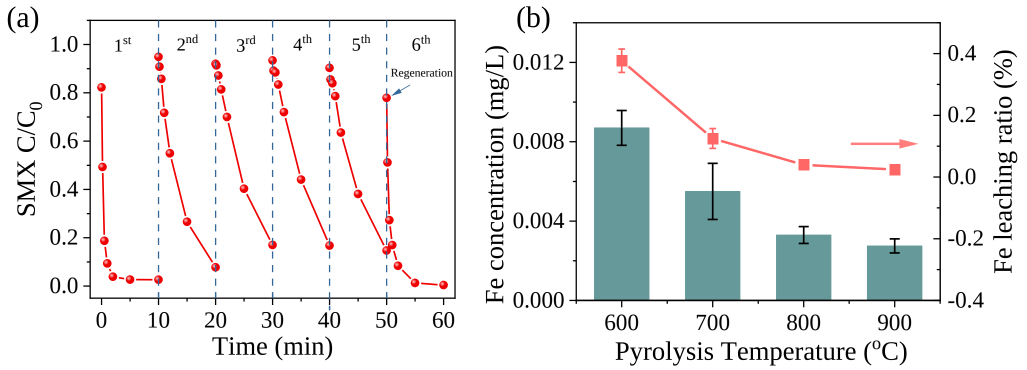

Figure S21. (a) Cycling tests of PMS activated Fe-SANs-800°C for the degradation of SMX, the annealing process at 800°C for 2 h was used to regenerate Fe-SANs-800°C; (b) The leaching of Fe ions during the PMS activated by Fe-SANs prepared at different pyrolysis temperatures. Reaction condition: [SMX] = 10 mg/L, [PMS] = 1 mmol/L, [Fe-SANs] = 100 mg/L, pH = 7.

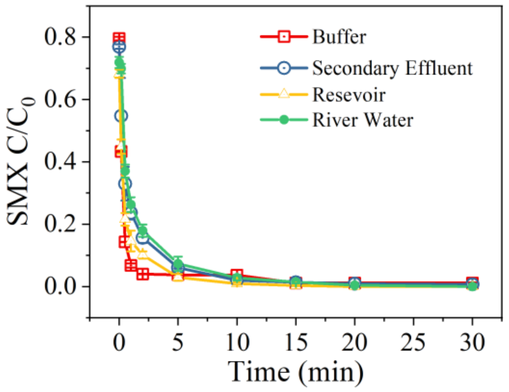

Figure S22. SMX degradation by the PMS activated Fe-SANs-800°C in the different actual surface water matrix. Reaction condition: [SMX] = 10 mg/L, [PMS] = 1 mmol/L, [Fe-SANs-800°C] = 100 mg/L, pH = 7 unless specified.

Table S1. HPLC test conditions for various micropollutants.

	Mobile phase			Flow rate	UV detector
	**Methanol**	**acetonitrile**	**0.1% formic acid**	**(mL/min)**	**(nm)**
BPA	-	40%	60%	0.8	273
CIP	-	20%	80%	0.5	277
SMX	40%	-	60%	0.8	263
CAP	40%	-	60%	0.8	278
CBZ	-	50	50% (H_2O)*	0.8	285
PNP	-	80%	20% (H_2O)*	0.5	318
PMSO	-	20%	80%	0.8	230
$PMSO_2$	-	80%	80%	0.8	215

*： The mobile phase of PNP was acetonitrile and H_2O, not 0.1% formic acid.

Table S2. Characteristics of three actual surface waters

	**DOC (mg-C/L)**	**TN (mg/L)**	**pH**	**TP (mg/L)**	**UV_{254}**
**SC effluent**	6.8±0.9	17.5±0.1	6.5	0.112±0.006	0.089±0.007
**River water**	8.4±1.2	14.9±2.0	6.8	0.064±0.001	0.134±0.009
**Reservoir water**	4.5±1.5	8.1±0.4	7.2	0.040±0.003	0.059±0.006

DOC: dissolved organic carbon

TN: Total nitrogen

TP: Total phosphorus

Table S3. EXAFS fitting parameters at the Fe K-edge for various samples

Sample	Shell	N^a	R(Å)^b	σ^2(Å^2)^c	ΔE_0(eV)^d	R factor
Fe foil	Fe-Fe	8	2.48	0.0053	6.9	0.0028
	Fe-Fe	6	2.85	0.0073		
Fe-SANs-600°C	Fe-N	5.4	2.02	0.0061	-4.7	0.0001
Fe-SANs-700°C	Fe-N	5.6	2.01	0.0066	-4.4	0.0001
Fe-SANs-800°C	Fe-N	5.5	2.01	0.0051	-5.6	0.0001
Fe-SANs-900°C	Fe-N	2.9	2.00	0.0066	-2.1	0.0003
	Fe-Fe	4.2	2.53	0.0075		

$^a N$: coordination numbers; $^b R$: bond distance; $^c\sigma^2$: Debye-Waller factors; ^{d} ΔE_0: the inner potential correction. R factor: goodness of fit. S_0^2 was set to 0.794, according to the experimental EXAFS fit of Fe foil by fixing CN as the known crystallographic value.

Table S4. Comparison of the pollutant degradation rates in different hyperoxide activation systems

Materials	Pollutant	pH	Concentration (mg/L)			k_{obs}	Ref.
			Peroxide*	catalyst	polluta	(min^{-1})	
FeCo-N-C	bisphenol A	3	74.1^{a}	100	20	1.25	[1]
Co-N-C	BP-4*	7.5	22.8^{a}	5	1.54	0.180	[2]
Fe-SiO_2	p-hydroxybenzoic acid	3	1000^{b}	100	20	0.200	[3]
Fe_1/CN	4-chlorophenol	3	114^{a}	500	12.9	0.55	[4]
Fe-N_4/C_3N_4	phenol	7	2618^{b}	500	200	0.510	[5]
Fe-N-C	bisphenol A	6.5	400^{a}	150	20	0.24	[6]
Fe-N-CNT	bisphenol A	3	45.6^{a}	20	11.4	6.140	[7]
Fe-N-C	2,4-	5.8	38.8^{c}	20	3.26	0.055	[8]
Fe-N-C	bisphenol A	3	400^{a}	200	25	0.104	[9]
FeN_5 SANs	Sulfamethoxazole	7	114^{a}	100	10	1.43	This

BP-4*: 5-benzoyl-4-hydroxy-2-ethoxybenzenesulfonic

*: ^{a} is peroxymonosulfate (PMS), ^{b} is hydrogen peroxide, ^{c} is peroxydisulfate (PDS).

SI Reference

[1] X. N. Li, X. Huang, S. B. Xi, S. Miao, J. Ding, W. Z. Cai, S. Liu, X. L. Yang, H. B. Yang, J. J. Gao, J. H. Wang, Y. Q. Huang, T. Zhang, B. Liu, *J. Am. Chem. Soc.* **2018**, *140*, 12469-12475.

[2] H. D. Xu, N. Jiang, D. Wang, L. H. Wang, Y. F. Song, Z. Q. Chen, J. Ma, T. Zhang, *Applied Catalysis B-Environmental* **2020**, *263*.

[3] Y. Yin, L. Shi, W. L. Li, X. N. Li, H. Wu, Z. M. Ao, W. J. Tian, S. M. Liu, S. B. Wang, H. Q. Sun, *Environ. Sci. Technol.* **2019**, *53*, 11391-11400.

[4] L. S. Zhang, X. H. Jiang, Z. A. Zhong, L. Tian, Q. Sun, Y. T. Cui, X. Lu, J. P. Zou, S. L. Luo, *Angew. Chem. Int. Ed. Engl.* **2021**, *60*, 21751-21755.

[5] S. F. An, G. H. Zhang, T. W. Wang, W. N. Zhan, K. Y. Li, C. S. Song, J. T. Miller, S. Miao, J. H. Wang, X. W. Guo, *Acs Nano* **2018**, *12*, 9441-9450.

[6] Y. Li, T. Yang, S. H. Qiu, W. Q. Lin, J. T. Yan, S. S. Fan, Q. Zhou, *Chem. Eng. J.* **2020**, *389*.

[7] K. Qian, H. Chen, W. L. Li, Z. M. Ao, Y. N. Wu, X. H. Guan, *Environ. Sci. Technol.* **2021**, *55*, 7034-7043.

[8] N. Jiang, H. D. Xu, L. H. Wang, J. Jiang, T. Zhang, *Environ. Sci. Technol.* **2020**, *54*, 14057-14065.

[9] Y. Gao, T. W. Wu, C. D. Yang, C. Ma, Z. Y. Zhao, Z. H. Wu, S. J. Cao, W. Geng, Y. Wang, Y. Y. Yao, Y. N. Zhang, C. Cheng, *Angew. Chem. Int. Ed. Engl.* **2021**, *60*, 22513-22521.
